# Serological and PCR-based markers of ocular *Chlamydia trachomatis* transmission in northern Ghana after elimination of trachoma as a public health problem

**DOI:** 10.1371/journal.pntd.0007027

**Published:** 2018-12-14

**Authors:** Laura G. Senyonjo, Oscar Debrah, Diana L. Martin, Adwoa Asante-Poku, Stephanie J. Migchelsen, Sarah Gwyn, Dzeidzom K. deSouza, Anthony W. Solomon, David Agyemang, Nana Biritwum-Kwadwo, Benjamin Marfo, Didier Bakajika, Ernest O. Mensah, Agatha Aboe, Joseph Koroma, James Addy, Robin Bailey

**Affiliations:** 1 Research Department, Sightsavers UK, Haywards Heath, United Kingdom; 2 Clinical Research Department, Faculty of Infectious and Tropical Diseases, London School of Hygiene & Tropical Medicine, London, United Kingdom; 3 Eye Care Unit, Ghana Health Service, Accra, Ghana; 4 Division of Parasitic Diseases and Malaria, Centers for Disease Control and Prevention, Atlanta, Georgia, United States of America; 5 Bacteriology Department, Noguchi Memorial Institute for Medical Research, University of Ghana, Accra, Ghana; 6 Parasitology Department, Noguchi Memorial Institute for Medical Research, University of Ghana, Accra, Ghana; 7 Sightsavers Ghana, Accra, Ghana; 8 Department of Neglected Tropical Diseases, Ghana Health Service, Accra, Ghana; 9 FHI360 Ghana, Accra, Ghana; King Saud University College of Medicine, SAUDI ARABIA

## Abstract

**Background:**

Validation of elimination of trachoma as a public health problem is based on clinical indicators, using the WHO simplified grading system. *Chlamydia trachomatis (Ct)* infection and anti-*Ct* antibody responses (anti-Pgp3) have both been evaluated as alternative indicators in settings with varying levels of trachoma. There is a need to evaluate the feasibility of using tests for *Ct* infection and anti-Pgp3 antibodies at scale in a trachoma-endemic country and to establish the added value of the data generated for understanding transmission dynamics in the peri-elimination setting.

**Methodology/Principal findings:**

Dried blood spots for serological testing and ocular swabs for *Ct* infection testing (taken from children aged 1–9 years) were integrated into the pre-validation trachoma surveys conducted in the Northern and Upper West regions of Ghana in 2015 and 2016. *Ct* infection was detected using the GeneXpert PCR platform and the presence of anti-Pgp3 antibodies was detected using both the ELISA assay and multiplex bead array (MBA). The overall mean cluster-summarised TF prevalence (the clinical indicator) was 0.8% (95% CI: 0.6–1.0) and *Ct* infection prevalence was 0.04% (95%CI: 0.00–0.12). Anti-Pgp3 seroprevalence using the ELISA was 5.5% (95% CI: 4.8–6.3) compared to 4.3% (95%CI: 3.7–4.9) using the MBA. There was strong evidence from both assays that seropositivity increased with age (p<0.001), although the seroconversion rate was estimated to be very low (between 1.2 to 1.3 yearly events per 100 children).

**Conclusions/Significance:**

Infection and serological data provide useful information to aid in understanding *Ct* transmission dynamics. Elimination of trachoma as a public health problem does not equate to the absence of ocular *Ct* infection nor cessation in acquisition of anti-*Ct* antibodies.

## Author summary

Trachoma is a disease caused by *Chlamydia trachomatis* (*Ct*). Validation of elimination of trachoma as a public health problem is based on clinical indicators. Antibody and infection data may provide a better understanding of transmission dynamics in elimination settings. Dried blood spots (DBSs) for antibody testing and ocular swabs for *Ct* infection testing were integrated into the pre-validation trachoma surveys conducted in the Northern and Upper West regions of Ghana in 2015 and 2016. *Ct* infection was detected using the GeneXpert PCR platform and the presence of anti-Ct antibodies were detected using both the ELISA and multiplex bead array (MBA). Very little infection was identified (0.04%). The conclusions from the ELISA and MBA testing were similar, with evidence of an association between increasing seroprevalence and age in 1-9-year olds. Infection and serological data provide useful insights into transmission dynamics. Even if an EU meets trachoma elimination targets, this may not reflect complete interruption of transmission of ocular *Ct* infection.

## Introduction

Trachoma is a disease caused by *Chlamydia trachomatis* (*Ct*). Repeated ocular infections [1] result in inflammation leading to conjunctival scarring, trichiasis (in-turned eyelashes which touch the eye) and ultimately corneal opacity (CO). The intervention strategy for trachoma is the World Health Organization (WHO)-endorsed SAFE strategy (S: Surgery for trachomatous trichiasis (TT); A: Antibiotics to clear *Ct* infection; F: Facial cleanliness and E: Environmental improvement to reduce transmission of *Ct*) [2,3]. Successful implementation of this strategy has resulted in a reduction in trachoma prevalence across many endemic countries [4–7]. WHO has set a goal of global elimination of trachoma as a public health problem by 2020 [8].

For trachoma elimination to be validated, countries must provide evidence that three criteria have been met. First, each previously-endemic evaluation unit (EU; populations of 100,000–250,000 people) must have reached and sustained, for at least two years, a prevalence of trachomatous inflammation—follicular (TF) in 1–9-year-olds of less than 5%. Second, each previously endemic EU must have reached a prevalence of TT previously unknown to the health system in ≥15-year-olds of less than 0.2%. Third, there must be an appropriately-resourced system to identify and manage incident trichiasis cases [9].

The WHO elimination thresholds for trachoma are based on clinical diagnostic indicators [9], using the simplified grading system [10]. However, TF has been shown to correlate poorly with *Ct* infection in low prevalence settings [11–14]. A follicular inflammatory response is known to persist for many weeks after infection has been cleared [15,16]. The presence of follicles deep to the upper tarsal conjunctiva is not a sign unique to *Ct* infection; a number of non-chlamydial pathogens including *Haemophilus influenzae* may elicit a similar response [14,17]. As such, the positive predictive value of a clinical diagnosis of TF for *Ct* infection can be reduced in low prevalence settings [18] where other aetiologies may account for a high proportion of TF. In the context of trachoma elimination, a lack of specificity of TF as an indicator will make it increasingly difficult to ensure that EUs are correctly categorised as endemic or not and that valuable resources are not wasted by unnecessarily prolonging interventions [19]. There are also concerns over the inter-grader agreement for diagnosis of TF, which becomes increasingly difficult to demonstrate [20] as trachoma prevalence decreases. As a result, there is a considerable interest in exploring whether and how alternative indicators could provide more objective evidence of elimination of trachoma as a public health problem, or be used as tools for post-validation surveillance [21].

Tests for anti-*Ct* antibody and *Ct* infection have been evaluated as alternative markers in settings with varying levels of trachoma [22–26]. In general, there has been very little or no *Ct* infection identified in areas where TF prevalence is below the elimination threshold [25–28]. Nucleic acid amplification tests (NAATs) including polymerase chain reaction (PCR) are highly specific and sensitive for ocular *Ct* infection [29,30]. The Cepheid GeneXpert platform is an automated, cartridge-based NAAT platform used widely across Africa for detection of *Mycobacterium tuberculosis* [31] that can detect *Ct* infection using different primers [29]. While a good test for *Ct* infection may have advantages over a proxy indicator, such as a sign of eyelid inflammation, collecting and analysing conjunctival swabs can be time-consuming, require specialist resources and personnel, and be potentially cost-prohibitive for national eye care or neglected tropical disease programmes [30]. The presence of anti-*Ct* antibodies, measured by multiplex bead array (MBA) [32,33], enzyme-linked immunosorbent assay (ELISA) [32,34,35] or lateral flow assay [32,36,37], may reflect cumulative exposure to *Ct* and when evaluated against age, represent transmission intensity over time [23,25,38,39]. Studies to date have predominantly focused on the detection of antibodies to Pgp3 [23,25,38], a conserved *Ct* plasmid protein found in both urogenital and ocular serovars [40]. The prevalence of anti-Pgp3 antibodies correlates fairly well with the prevalence of TF [22,23,25,26,38,39]. In post-elimination settings, the prevalence of Pgp3 seropositivity in children has shown either no increase with age or only minimal increases with increasing age [22,25,26,38].

The feasibility of generating district-level data for *Ct* infection and anti-*Ct* antibodies and how to interpret them for programmatic decision-making is still to be determined. A better understanding of the age-prevalence profiles in the post-elimination setting is also needed [9]. In 2015–2016, Ghana conducted a set of population-based surveillance surveys that demonstrated that all EUs previously endemic for trachoma had maintained the elimination threshold of <5% TF in the absence of large scale antibiotic treatment [41]. We integrated ocular swabs and DBSs into the surveys, providing an opportunity to evaluate the feasibility of using tests for *Ct* infection and anti-Pgp3 antibodies at scale in a trachoma-endemic country. We also compared antibody data collected by ELISA in Ghana to MBA data run at the Centers for Disease Control and Prevention (CDC), USA. These data were also used to evaluate whether measures of infection or Pgp3 antibody response have added value for understanding transmission dynamics in the peri-elimination setting.

## Methods

### Ethics statement

The study was approved by the Ghana Health Service (GHS) Ethics Review Committee (Reference GHS-ERC: 03/07/15) and the London School of Hygiene & Tropical Medicine Research Ethics Committee (Reference 10285). CDC involvement was determined not to constitute engagement in human subjects research, as CDC staff had no interaction with study participants.

Written informed consent was sought from caregivers of all children who participated in this study. Children who were able to provided verbal assent. Individuals with active trachoma were given 1% tetracycline eye ointment.

### Study area

**Fig 1:**
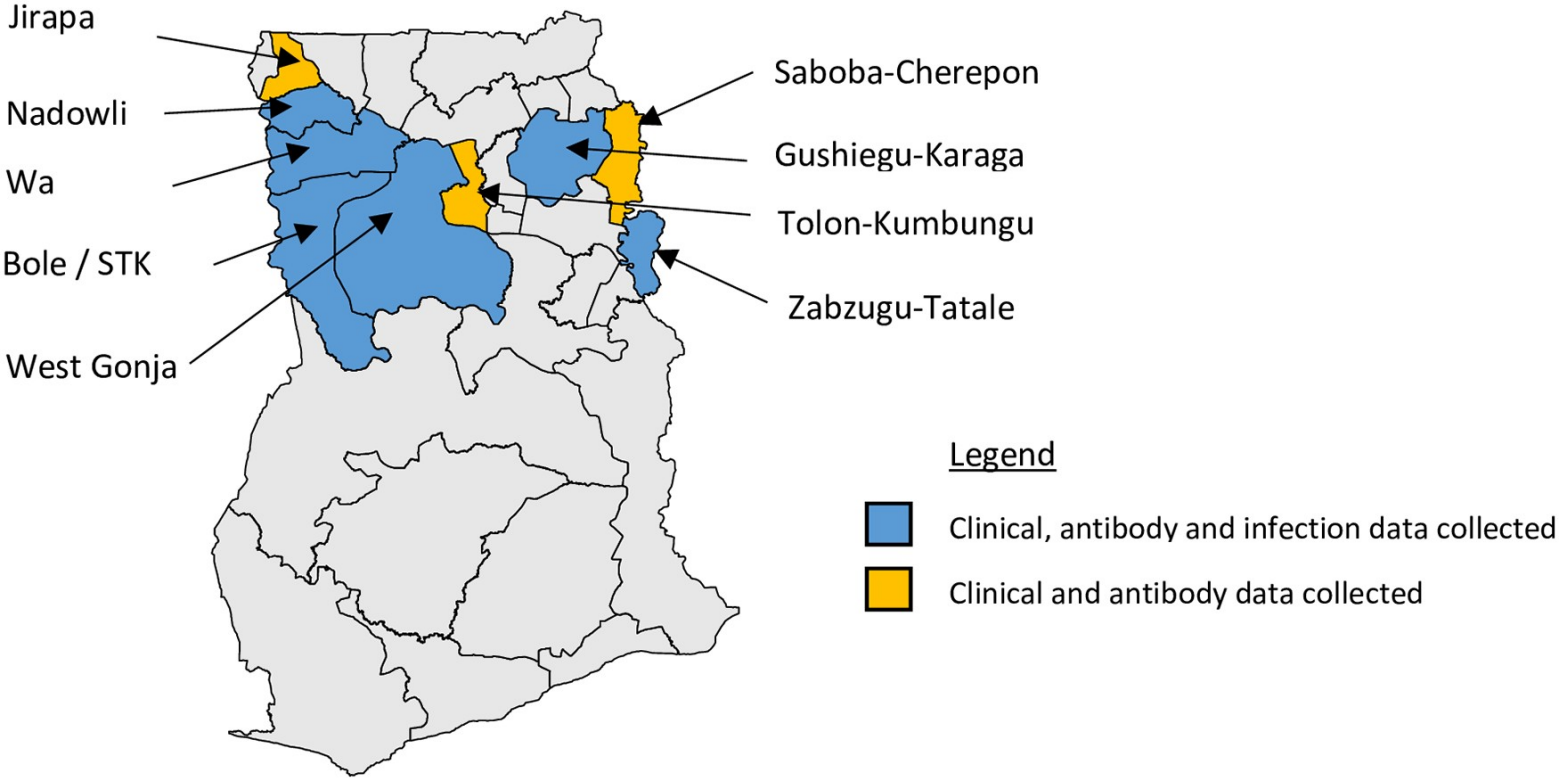
Map of the evaluation units, indicating data types generated in each unit. Adapted from an open source map retrieved from www.mapmaker.com.

The study was conducted in the Northern and Upper West regions of Ghana, Fig 1. Surveys were conducted between November 2015 and April 2016.

Baseline assessments of trachoma prevalence were conducted in all 18 EUs between 1999 and 2003 [41]. A total of four EUs had a TF prevalence (in some cases combined TF/TI prevalence) of above 10% in children aged 1–5 years, five had a prevalence of 5–9.9% and nine had a prevalence of less than 5%. Based on WHO recommendations [42], GHS implemented the SAFE strategy, delivering EU-wide mass drug administration (MDA) of azithromycin in the EUs with TF prevalence >10% and antibiotic distribution targeted at community level in the EUs with TF prevalence <10%).

In 2008, impact surveys were conducted and all 18 EUs were declared to have reached or maintained the TF elimination threshold [43]. In 2011, GHS implemented a surveillance strategy that involved annual community and school screening for detection of TF and TT [41]. Eight communities identified during impact surveys or surveillance to have TF ≥5% were given three years of azithromycin MDA.

### Survey design

A series of two-stage cluster-sampled population-based surveys were conducted in all 18 EUs as part of the Ghana pre-validation surveillance process [41]. A sub-set of nine EUs had additional indicators collected and evaluated, the results of which are the focus of this paper. Clinical, antibody and infection data were collected from six EUs (Table 1 and Fig 1). An additional three EUs were sampled for clinical and antibody data only (Table 1 and Fig 1). The EUs selected were chosen to represent a range of baseline TF prevalence and provide geographical spread. Infection data were not collected from the additional three EUs because of financial and time constraints related to the analysis.

**Table 1:** Number of samples analysed by evaluation unit.

Sample	Evaluation Unit									
	Bole/ Sawla-Tuna-Kalpa	West Gonja	Zabzugu-Tatale	Tolon Kumbungu	Gushiegu Karaga	Saboba Cherepon	Wa	Nadowli	Jirapa	Total
**DBS and TF**	1,307	1,148	1,345	1,361	1,367	1,202	1,357	1,363	1,280	**11,730**
**Ocular swabs**	1,312	925	1,349	ND	1,372	ND	1,364	1,366	ND	**7,688**

DBS: dried blood spot; ND: Not done; TF: trachomatous inflammation—follicular

The primary sampling unit was a community (village), selected with probability proportional to population size, and the secondary sampling unit was the household, selected using compact segment sampling. All children aged between one and nine years residing in the selected households were eligible for inclusion. The sample size calculations and sampling criteria were based on TF parameters and have been detailed elsewhere [41].

Survey data were collected electronically using a secure Open Data Kit-based Android smartphone application (LINKS, Task Force for Global Health, Atlanta, GA, USA; https://linkssystem.org) [44]. Data were uploaded to a cloud-located server with password-protected access only available to identified study investigators.

### Clinical assessment

All graders were certified using Global Trachoma Mapping Project (GTMP) methodologies, described elsewhere [20]. Due to the expected low prevalence of trachoma in Ghana, the graders were trained and certified by examining children in Sokoto, Nigeria, where a number of districts still have TF prevalence estimates above the elimination threshold [7]. Each grader had to achieve a minimum kappa score of 0.7 for TF in an inter-grader agreement test with a grader trainer who had been certified by the GTMP.

Children aged 1–9 years were assessed for all five signs of trachoma (TF, trachomatous inflammation–intense (TI), trachomatous scarring (TS), TT and CO) as per the WHO simplified grading criteria [10].

### Sample collection

Ocular swabs for infection testing were collected by passing a dry sterile polyester-tipped swab horizontally along the upper tarsal conjunctiva of the left eye, at least three times, rotating the shaft 120° with each pass. Control procedures were put into place to avoid field contamination, in particular washing hands at each new household, changing gloves between each examinee, and ensuring the end of the swab, once it had touched the conjunctiva, was placed directly into and broken off within a tube, which was sealed without further swab contact. Negative controls were taken after every 50 swabs by passing a clean swab in the air within five centimetres of a child’s eyes. Collected swabs were kept cool in the field and then refrigerated at 4°C for up to one week before being shipped on ice packs to Noguchi Memorial Institute for Medical Research (NMIMR) in Accra, where they were stored at -20°C until the time for analysis. Specimens were limited to one freeze/thaw cycle to reduce potential DNA degradation [29].

Dried blood spots (DBSs) were collected for serological testing. After cleaning with an alcohol-soaked swab, the participant’s finger was pricked using a sterile single-use lancet and the blood collected directly onto filter paper (Trop-Bio, Townsville, Australia). The filter paper had six projections, each calibrated to collect 10 μL of blood. The filter papers were air-dried in the shade then individually packed in sealable plastic bags and stored in a larger (gallon-size) sealable plastic bag with desiccant. DBSs were refrigerated at 4°C for up to one week before being shipped at ambient temperature to Accra and stored at -20°C until analysis.

### Nucleic acid and antibody testing

PCR and ELISA were done at NMIMR in Accra, Ghana. MBA analysis was performed at the CDC in Atlanta, USA.

Ocular swabs were analysed for the presence of *Ct* DNA using the GeneXpert IV machine (Cepheid, Sunnyvale, USA) and GeneXpert CT/NG Assay (Cepheid, Sunnyvale, USA). Swabs were eluted using sterile diethylpirocarbonate (DEPC) water and pooled into groups of five samples as per a published pooling strategy [45]. The individual samples that made up a positive pool were tested separately to identify the positive sample(s). Results were reported as *Ct*-positive, negative or indeterminate (invalid, error or no result). The GeneXpert can produce an invalid result if there is failure of the sample adequacy control, which requires human DNA in the sample, or specimen processing control, indicating that amplification was inhibited [30]. Indeterminate pools were re-tested using a new aliquot of the specimen and a new cartridge. Control swabs collected in the field were analysed individually. Two *Ct* positive and two *Ct* negative processing controls were run at the beginning of each week.

DBSs were tested for antibodies to the *Ct* antigen Pgp3 using the semi-quantitative ELISA assay, described elsewhere [32,34]. Briefly, Immulon 2HB 96-well plates (ThermoFisher Scientific, Waltham, MA) were sensitized with 50 μL of Pgp3 antigen (500 ng/mL concentration) overnight at 4°C. DBSs and serum samples were diluted 1:50 in PBS containing 0.3% Tween-20 and 5% milk powder (PBST-milk) and stored overnight at 4°C. The next day, wells were washed with PBST (0.3% Tween-20 in PBS) and then blocked with PBST for one hour. Sample (50 μL) was added to wells and incubated for two hours at room temperature. Wells were then washed with PBST and incubated with 50 μL anti-human IgG conjugated to horseradish peroxidase (HRP) (1:10,000 dilution) (Southern Biotech, Birmingham, AL) to detect bound antibody. After four washes with PBST, 50 μL of 3, 3′, 5, 5′-tetramethylbenzidine (TMB) developing reagent (KPL, Gaithersburg, MD) was added to the wells and the reaction was stopped with 50 μL 1N H_2_SO_4_ after the predetermined interval. The optical density (OD) at 450 nm was read using an ELx808 Absorbance Microplate Reader (Biotek, Winooski, USA). OD values were corrected for background absorbance by subtracting the average OD of the two wells containing PBST-milk. The blanked OD values for all samples and controls were then normalised against the 200 U standard included on the same plate [34].

For the MBA, samples were tested in single-wells with Pgp3-coupled beads, as previously described [46,47]. Briefly, one DBS extension was diluted 1:320 in PBS containing 0.5% casein, 0.3% Tween 20, 0.5% polyvinyl alcohol, 0.8% polyvinylpyrrolidone, 0.02% sodium azide and 3 μg/mL *E*. *coli* extract (Buffer B). Coupled beads (2500 per antigen) were incubated with 40 μL of diluted sample per well in a 96-well filter plate (Millipore, Billerica, MA) for 1.5 hours. Wells were washed three times with PBS containing 0.05% Tween 20 (PBST2) and incubated with 50 ng biotinylated mouse anti-human IgG (Southern Biotech, Birmingham, AL) and 20 ng biotinylated mouse anti-human IgG4 (Southern BioTech) for 45 minutes to detect bound antibody. Wells were washed three times with PBST2 and incubated with 250 ng phycoerythrin-labelled streptavidin (Invitrogen, South San Francisco, CA) for 30 minutes to detect bound secondary antibody. After three washes with PBST2, wells were incubated for 30 minutes with 0.5% BSA, 0.05% Tween 20, 0.02% sodium azide in PBS to remove any non-specific binding. After one wash, wells were suspended in 125 μL of PBS and read on a Bio-Plex 200 instrument (Bio-Rad, Hercules, CA) equipped with Bio-Plex manager 6.0 software (Bio-Rad). The median fluorescence intensity (MFI) with the background from the blank well (Buffer B alone) subtracted out (MFI-bg) was recorded for each antigen for each sample. All samples were analysed masked to demographic and examination findings.

### Statistical analysis

Only individuals with complete serological, infection and clinical data (or serological and clinical data, in EUs where ocular swabs were not collected) were included in the analysis. The dataset was presumed to be self-weighted but the analysis was adjusted (using STATA’s svy command) for the cluster sampling methodology.

Individuals were classified as seropositive or seronegative based on normalised OD values on the ELISA platform, and on the MFI-bg, after a log (x + 1) transformation, for the MBA. This transformation took into account that the MFI-bg included values of zero. The seropositive cut-off was defined using a finite mixture model based on maximum likelihood methods [34], with the threshold for seropositivity set as the mean of the Gaussian distribution of the seronegative population, plus four standard deviations [38].

To examine force of infection (FoI), the rate at which susceptible individuals acquire infection, the seroconversion rate (SCR), the rate at which seronegative individuals become seropositive, was estimated using a simple reversible catalytic model (RCM) fitted to seroprevalence in yearly age groups, using maximum likelihood estimates. Evidence for a change in SCR over time was explored by comparing two models using the profile likelihood method; the first model assumed constant transmission over time and the second assumed a potential change in the FoI at a specified time point [48].

Statistical analysis was conducted using R 3.4.0 [49] and STATA 12.0 [50]. The data were adjusted for age and gender based on the Ghana 2010 census [51]. The adjusted cluster-summarised mean prevalence was calculated for all the data and at the level of the EU. Bootstrap estimation was used to determine confidence intervals around prevalence estimates, based on 10,000 iterations and taking the 2.5^th^ and 97.5^th^ centiles.

For the serology data, chi-square tests were used to determine univariate associations. The non-parametric test for trend was used to determine an increase in seropositivity with age. Positive univariate associations of seropositivity at the individual level (age, EU, baseline TF endemicity) and gender (included a priori) were included in multivariate logistic regression models. The likelihood ratio test was used to determine the model of best fit. Regression was used for analysis of associations between continuous variables at the level of the EU. The geometric mean antibody titre was calculated using a log (x+1) transformation to take into account zero values.

A measure of cluster-level heterogeneity was determined by calculating the intra-cluster correlation coefficient (ICC) and design effect (DE). The ICC reveals how strongly observations in the same cluster resemble each other. The DE is the ratio of the variance in the collection of observations amassed using cluster sampling to the variance assuming the same sample size had been generated using simple random sampling [52]. The ICC and DE for this dataset were determined using STATA.

## Results

### Demographic information

Overall 96.0% of children resident in the selected households were examined, 3.6% were absent at the time of the survey and 0.4% refused to participate. A total of 11,730 DBSs were collected across nine districts, analysed by ELISA and matched to demographic and clinical data, Table 1. A total of 10,902 DBSs were also analysed by MBA. The number of samples taken by age group are detailed in Table 2.

**Table 2:** Number of samples analysed by age.

Sample	Age (years)									
	1	2	3	4	5	6	7	8	9	Total
**DBS and TF**	833	1,361	1,652	1,638	1,650	1,448	1,148	983	1,017	**11,730**
**Ocular swabs**	556	894	1,057	1,092	1,073	957	756	640	663	**7,688**

DBS: dried blood spot; TF: trachomatous inflammation—follicular

A total of 7,688 ocular swabs were taken across six districts, analysed and matched to demographic and clinical data. Overall, 50.2% of individuals surveyed were male and the median age was 5 years old.

### Clinical data

Across all EUs, 1.0% of individuals (n = 112; 95%CI: 0.8–1.2) had TF in one or both eyes; of those, 67.9% (n = 76) had bilateral TF. There was no evidence of an association between TF and age (z = -0.48; p = 0.63). No TT was identified in children. The median age of individuals with TF was 4.5 years and 53.6% were female.

The overall cluster-summarised mean TF prevalence was 0.8% (95%CI:0.6–1.0), with an EU-level range of 0.5–1.1%, Table 3.

**Table 3:** Cluster-summarised mean prevalence of trachomatous inflammation—follicular (TF), *Chlamydia trachomatis* (*Ct*) infection and Pgp3 seropositivity by ELISA and MBA in children aged 1–9 years across selected evaluation units in Northern and Upper West regions of Ghana.

Indicator		Evaluation unit									
		Northern Region						Upper West Region			
		Bole/ Sawla-Tuna-Kalpa (STK)	West Gonja	Zabzugu-Tatale	Tolon Kumbungu	Gushiegu Karaga	Saboba Cherepon	Wa	Nadowli	Jirapa	Total
**Baseline TF prevalence**		5–9.9%	≥10%	5–9.9%	≥10%	<5%	<5%	≥10%	<5%	5–9.9%	NA
**TF**	%	0.5	1.1	1.0	0.8	0.7	0.8	1.1	0.8	0.5	0.8
	95%CI (n)	0.2–1.0 (9)	0.4–1.2 (14)	0.5–1.5 (18)	0.4–1.3 (15)	0.3–1.1 (12)	0.3–1.3 (9)	0.5–1.9 (13)	0.3–1.5 (12)	0.1–1.0 (10)	0.6–1.0 (112)
	DE	1.61	1.27	2.88	0.80	0.86	0.86	1.11	1.98	1.68	1.81
***Ct* infection**	%	0.07	0.0	0.2	ND	0.0	ND	0.0	0.0	ND	0.04
	95%CI (n)	0.00–0.12 (1)	0.0–0.0 (0)	0.0–0.6 (3)	ND	0.0–0.0 (0)	ND	0.0–0.0 (0)	0.0–0.0 (0)	ND	0.0–0.1 (4)
	DE	1.01	1 (NA)	3.01	ND	1 (NA)	ND	1 (NA)	1 (NA)	ND	2.49
**Seropositive by ELISA (anti-Pgp3 antibodies)**	%	8.2	3.9	7.2	2.5	4.0	6.1	7.4	5.0	5.9	5.5
	95%CI (n)	5.7–11.1 (100)	2.4–5.4 (47)	4.3–10.6 (90)	1.5–3.6 (37)	2.3–6.1 (56)	4.3–8.6 (74)	5.0–10.1 (105)	3.8–6.2 (71)	4.0–8.3 (82)	4.8–6.3 (662)
	DE	1.97	2.19	4.25	1.32	2.21	1.76	2.98	0.92	1.74	2.49
**Seropositive by MBA (anti-Pgp3 antibodies)**	%	8.4	3.9	5.5	2.0	3.0	3.2	6.4	2.8	3.7	4.3
	95%CI (n)	6.3–10.6 (90)	2.3–5.7 (39)	3.3–8.3 (66)	1.1–3.1 (25)	1.8–4.5 (39)	2.1–4.6 (37)	4.1–8.9 (61)	1.9–3.8 (39)	2.3–5.3 (48)	3.7–4.9 (444)
	DE	2.23	1.17	3.75	1.50	1.77	0.72	2.20	1.37	1.68	2.30

Cluster-summarised mean prevalence reported, except for baseline TF prevalence

ND: Not done NA: Not applicable DE: Design effect

### Infection data

Four infections were identified, giving a cluster-summarised mean prevalence of 0.04% (95%CI:0.00–0.12), Table 3. The four samples were from two different clusters, one from a community in Bole and the other three from separate households of a single community in Zabzugu-Tatale. Both of these EUs had baseline TF prevalences of 5–9.9% in children aged 1–5 years. The median age of those infected was 6.5 years.

A total of 83 samples (1.1%) had indeterminate PCR results. All control swabs were negative for *Ct* DNA.

### Serological data

The seropositive cut-off (four standard deviations from the mean of the seronegative population) for the ELISA was 1.091 OD_450nm_ and for the MBA was 5.188 for the log of the MFI-bg.

The overall cluster-summarised mean seroprevalence was 5.5% (95%CI: 4.8–6.3) by ELISA and 4.3% (95%CI: 3.7–4.9) by MBA. Pgp3 seropositivity by ELISA differed by EU (F stat = 3.61; p = 0.001), with the highest seroprevalence in Bole/Sawla-Tuna-Kalpa (8.2% 95%CI: 5.7–11.1) and lowest in Tolon-Kumbungu (2.5%; 95%CI: 1.5–3.6) (Table 3). This pattern in EU seropositivity was also reflected by the MBA results.

**Fig 2:**
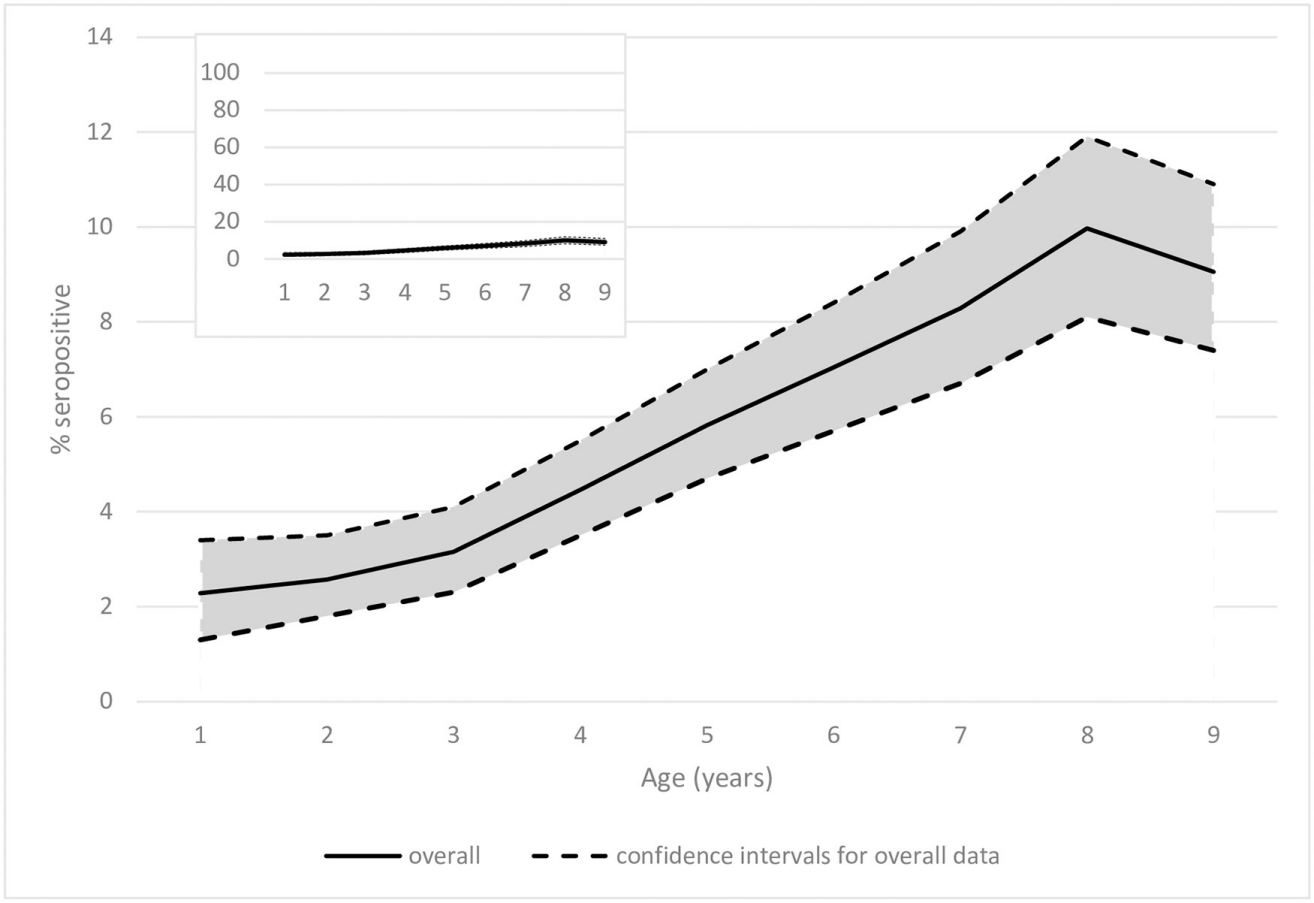
Seropositivity by age for the ELISA platform: Overall data 95%CI envelopes; inset graph shows the overall seropositivity by age using a 100% y-axis scale.

Seropositivity increased with age; this association held when analysing results from either platform (p<0.001) (Fig 2).

The model of best fit for the RCM was a singular SCR for the time period studied. Overall, there was a low seroconversion rate of 1.3 yearly events per 100 children (95%CI: 1.1–1.6), using the ELISA data. A similarly low SCR was reported using MBA data, with 1.2 yearly events per 100 children (95% CI: 0.9–1.6).

Pgp3 seropositivity was also associated with baseline EU-level TF prevalence (p<0.001) after controlling for potential confounders. The highest proportions of seropositive children were in those EUs that had a baseline TF prevalence of 5–9.9% in children aged 1–9 years. In the multi-variate model, there was an interaction observed between age and baseline TF prevalence: EUs with lower baseline TF prevalence estimates had comparatively greater odds of having older children who were seropositive by ELISA as opposed to younger children (p<0.001) (Table 4). Analysis of the seropositivity data generated by the MBA resulted in the same conclusions.

**Table 4:** Odds ratios for anti-Pgp3 seropositivity by ELISA, by evaluation unit-level baseline trachomatous inflammation—follicular (TF) prevalence and age of the individual, accounting for gender and EU.

TF prevalence at baseline	Age group (comparator age: 1–3 years)	
	4–6 years	7–9 years
<5%	3.4	4.4
5–9.9%	1.7	3.1
>10%	2.3	3.5

The overall geometric mean of normalised ODs was 0.49 for the ELISA and 13.58 MFI-bg for the MBA platform. The geometric mean antibody titre increased with age (p<0.001). The strongest antibody responses (top 10% antibody titres of all seropositive individuals) were detected in children from the EUs with the highest seroprevalence estimates (for ELISA data: R^2^ = 0.68, p = 0.006).

### Heterogeneity of data

An analysis of heterogeneity of data for the three indicators suggests some variability within clusters (Table 3). The overall DE for clinical data was quite low at 1.81 (EU-level range 0.80–2.88) and an ICC of 0.01 (cluster-level TF prevalence range 0–7.1%). The DE was higher for serologic data, 2.49 for the ELISA (2.30 for MBA data) with a corresponding ICC of 0.03 (cluster-level seroprevalence range 0–32.1%). There was variation in DE across EUs, ranging from 0.92 to 4.25 for the ELISA data and 0.72 to 3.75 for the MBA data, with the highest values in Zabzugu-Tatale. In the two clusters that were found to have infection, there was also high seropositivity (>15% seroprevalence using the ELISA and MBA). The high heterogeneity of the serology data (in Zabzugu-Tatale) was largely driven by one cluster that had infection (5.4%) and the highest proportion of seropositive individuals (32.1%). After removing that cluster from the DE calculations, the DE for (ELISA) serology dropped from 4.25 to 1.97 (3.75 to 0.90 for the MBA data). In EUs where infection was detected the DE was 3.01 (Zabzugu-Tatale) and 1.01 (Bole/Sawla-Tuna-Kalpa).

## Discussion

Ghana has met the active trachoma criterion for elimination of trachoma as a public health problem, a measure based on TF parameters [41,53]. The collection of alternative indicators in pre-validation surveillance surveys allowed us to generate a more complete understanding of transmission dynamics in this setting. As evidenced in this study, elimination of trachoma as a public health problem does not equate to the absence of ocular *Ct* infection nor cessation in acquisition of anti-*Ct* antibodies. There are a number of potential explanations for this finding.

Infection was detected at very low levels (0.04%) and from limited sites, similar to findings from other studies in analogous settings which found no or very low prevalence of *Ct* [18,23,26,27]. These infection cases could be false positives, given the specificity of the assay [29,30,45], or a result of cross-contamination in the swab collection or analysis process. Equally, we cannot rule out the possibility that these were urogenital strains, acquired, for example, by transfer to children’s eyes, as a result of poor parental hand hygiene. However, the triangulation of serological, clinical and infection data in the communities suggests these are true *Ct* cases, whether ocular or urogenital strains. A cross-sectional study cannot tell us whether infection in these communities is transient or it is persistent and a potential risk for recrudescence. It is necessary to follow-up these select communities to determine if there is evidence of continued ocular *Ct* transmission over time and provide an opportunity to evaluate a model for post-validation surveillance.

Pooling of ocular swab samples was used in this study, a process known to be particularly cost-efficient in settings with low infection prevalence [45,54–56]. However, there are some concerns pooling can reduce the sensitivity of the test. Evidence suggests the impact would be minimal and likely to affect those individuals with lower ocular bacterial load [57], who are likely to be less important as drivers of transmission [58,59]. A slight loss in sensitivity would be tolerable where programmatic decision-making relied on EU-level classification; the specificity of the test, however, is critical. The GeneXpert machine is a relatively simple, mobile platform which is closed and self-contained, minimising potential cross-contamination [30], and was successfully used in Ghana. However, even with a simple platform to use and with pooling of samples for analysis, a key limitation of routinely including tests for infection in programmatic surveys will be the time and cost required to analyse the samples using NAATs.

Overall seroprevalence in children aged 1–9 years was similar here to estimates reported for other elimination or post-intervention settings [25,38]. The seroprevalence data by age allows estimation of transmission intensity over time. In Ghana there was evidence of a statistically significant increase in seropositivity with age (p<0.001). Studies in post-MDA settings in Tanzania and The Gambia found a similar increase in seropositivity with age [25,38]. Other studies in settings where elimination thresholds have been sustained over a number of years have found no association of seropositivity with age [22,26], which may be a reflection of interruption of transmission but also potentially a lack of power to be able to detect low rates of seroconversion. The data from Ghana suggested a history of low level ongoing seroconversion (1.2 to 1.3 events per 100 children per year) across the Northern and Upper West regions. Ghana stopped district-level antibiotic MDA at least eight years before the time of this study, and the single SCR reported likely reflects this, as any significant change in SCR would probably have occurred before most children enrolled in this study were born. Therefore, the SCR likely reflects a stable FoI in a post-elimination setting. These findings reinforce the idea that very low levels of on-going *Ct* transmission are not inconsistent with trachoma elimination as a public health problem. As some estimates suggest that more than 150 infections are needed over a lifetime to develop TT [1], the level of transmission we estimated in Ghana is highly unlikely to be of public health concern.

It is difficult to directly compare the seropositivity rates across studies because there is currently no agreed standard methodology for defining the threshold used to determine seropositivity [34]. We used an internally calibrated approach, which has the advantage that it does not rely on external positive or negative controls from different populations. This methodology could artificially define a seronegative and seropositive group [60] and inflate the number of “low intensity” positives. We used a conservative cut-off threshold of four standard deviations from the mean of the seronegative population to increase specificity and reduce likelihood of this outcome. Another difficulty in interpreting the serological data is that antibodies to Pgp3 do not distinguish between urogenital and ocular infection, and *Ct* exposure could have occurred at birth through ocular or respiratory infection from a mother with genital *Ct* [61]. This is an important consideration, however, although there is a paucity of data on the prevalence of sexually transmitted infections in Ghana, where it is documented it is reported to be relatively low [62–64] and urogenital *Ct* infection is not believed to be high in the Northern and Upper West regions of the country. If vertical transmission was a major driver of antibody acquisition, then it could be expected that the gradient of the age seroprevalence curve would be flat or even negative with increasing age [38].

It is noted that the use of an RCM is a simplification of real-world transmission dynamics, and using data from a single cross-sectional study to determine two linked RCM parameters is problematic. However, the SCR estimated here is similar to that generated in other studies in comparable environments [38]. Longitudinal data would help to generate better estimates of SCR [65].

In this study, we compared data obtained from the Pgp3 ELISA run in-country to MBA data generated at CDC. While the overall prevalences differed, the qualitative conclusions drawn from the data did not change and in particular the SCR estimates from the two methodologies did not differ significantly. While quantitative data collected on different platforms are difficult to compare, the SCR data presented here suggest a robustness of anti-Pgp3 antibody data. ELISA is relatively inexpensive and the protocol has been shown to be implemented effectively in trachoma-endemic countries. A key advantage of the MBA platform is that it allows for integrated serosurveillance of multiple pathogens [66], however it is unlikely to be widely available in the near future in trachoma-endemic countries, many of which are reluctant to export biological samples for analysis. A rapid test such as a lateral flow assay [36] might be the best format for application to trachoma elimination.

Infection and in particular serological data provide useful insights into transmission dynamics. Even if an EU meets trachoma elimination targets, this may not reflect complete interruption of transmission of *Ct* infection.

## Supporting information

S1 Checklist STROBE checklist. (DOCX)
