# Supplementary material for: Serological and PCR-based markers of ocular Chlamydia trachomatis transmission in northern Ghana after elimination of trachoma as a public health problem
Source: PLoS Negl Trop Dis. 2018 Dec 14;12(12):e0007027. doi: 10.1371/journal.pntd.0007027 (PMC6310292; doi:10.1371/journal.pntd.0007027)
Supplement: S1 Checklist — (DOCX) [file pntd.0007027.s001.docx]

STROBE Statement—checklist of items that should be included in reports of observational studies

|  | Item No. | Recommendation | Page  No. | Relevant text from manuscript |
| --- | --- | --- | --- | --- |
| **Title and abstract** | 1 | (*a*) Indicate the study’s design with a commonly used term in the title or the abstract | 1 | Title; Abstract, methods sub-section |
|  |  | (*b*) Provide in the abstract an informative and balanced summary of what was done and what was found | 1-2 | Abstract, methods and conclusions/significance sub-section |
| Introduction | | | |  |
| Background/rationale | 2 | Explain the scientific background and rationale for the investigation being reported | 5 | Introduction, para 1 of page 5: The feasibility of generating district-level data for Ct infection and anti-Ct antibodies and how to interpret it for programmatic decision-making is still to be determined. A better understanding of the age-prevalence profiles in the post-elimination setting is also needed |
| Objectives | 3 | State specific objectives, including any prespecified hypotheses | 5 | Introduction, para 1 of page 5: We integrated ocular swabs and DBSs into the surveys, providing an opportunity to evaluate the feasibility of using tests for Ct infection and anti-Pgp3 antibodies at scale in a trachoma-endemic country. We also compared antibody data collected by ELISA in Ghana to MBA data run at the Centers for Disease Control and Prevention (CDC), USA. These data were also used to evaluate whether measures of infection or Pgp3 antibody response have added value for understanding transmission dynamics in the peri-elimination setting. |
| Methods | | | |  |
| Study design | 4 | Present key elements of study design early in the paper | 6-7 | Survey design sub-section: A series of two-stage cluster-sampled population-based surveys were conducted in all 18 EUs as part of the Ghana pre-validation surveillance process. A sub-set of nine EUs had additional indicators collected and evaluated, the results of which are the focus of this paper. Clinical, antibody and infection data were collected from six EUs. |
| Setting | 5 | Describe the setting, locations, and relevant dates, including periods of recruitment, exposure, follow-up, and data collection | 5 | Study area sub-section of methods pg 5: Overview of area given and map (figure 1), dates of survey |
| Participants | 6 | (*a*) *Cohort study*—Give the eligibility criteria, and the sources and methods of selection of participants. Describe methods of follow-up  *Case-control study*—Give the eligibility criteria, and the sources and methods of case ascertainment and control selection. Give the rationale for the choice of cases and controls  *Cross-sectional study*—Give the eligibility criteria, and the sources and methods of selection of participants | 6 | Methods, sampling process sub-section; Sampling process used – psu – community selected probability proportional to size and ssu household selected using compact segment sampling |
|  |  | (*b*) *Cohort study*—For matched studies, give matching criteria and number of exposed and unexposed  *Case-control study*—For matched studies, give matching criteria and the number of controls per case | NA |  |
| Variables | 7 | Clearly define all outcomes, exposures, predictors, potential confounders, and effect modifiers. Give diagnostic criteria, if applicable | 7  8  9 | Methods, clinical assessment sub-section: Clinical examination using Thylefors simplified grading system  Methods, nucleic acid and antibody testing sub-section: Ocular swabs were analysed for the presence of Ct DNA  Methods, 2^nd^ para page 9: DBSs were tested for antibodies to the Ct antigen Pgp3  Further information given on statistics analysis |
| Data sources/ measurement | 8* | For each variable of interest, give sources of data and details of methods of assessment (measurement). Describe comparability of assessment methods if there is more than one group | 7-9 | Methods, nucleic acid and antibody testing sub-section: Details given on sample collection |
| Bias | 9 | Describe any efforts to address potential sources of bias | 6 | Methods, survey design sub-section, para 2: Methodologies for selecting participants were outlined |
| Study size | 10 | Explain how the study size was arrived at | 7 | Methods, para 1, pg 7: Referred to sample size calculations as published elsewhere |

Continued on next page

| Quantitative variables | 11 | Explain how quantitative variables were handled in the analyses. If applicable, describe which groupings were chosen and why | 10-12 | Methods, statistical analysis sub-section: Explanations of analysis given |
| --- | --- | --- | --- | --- |
| Statistical methods | 12 | (*a*) Describe all statistical methods, including those used to control for confounding | 11 | Methods, para 3 of pg 11: Multivariate logistic regression |
|  |  | (*b*) Describe any methods used to examine subgroups and interactions | 11 | Methods, para 3 of pg 11: For the serology data, chi-square tests were used to determine univariate associations. The non-parametric test for trend was used to determine an increase in seropositivity with age. Positive univariate associations of seropositivity at the individual level (age, EU, baseline TF endemicity) and gender (included a priori) were included in multivariate logistic regression models. The likelihood ratio test was used to determine the model of best fit. Regression was used for analysis of associations between continuous variables at the level of the EU. The geometric mean antibody titre was calculated using a log (x+1) transformation to take into account zero values. |
|  |  | (*c*) Explain how missing data were addressed | 10 | Methods, statistical analysis sub-section, para 1: Only individuals with complete serological, infection and clinical data were included in the analysis. |
|  |  | (*d*) *Cohort study*—If applicable, explain how loss to follow-up was addressed  *Case-control study*—If applicable, explain how matching of cases and controls was addressed  *Cross-sectional study*—If applicable, describe analytical methods taking account of sampling strategy | 10 | Methods, statistical analysis sub-section, para 1: The dataset was presumed to be self-weighted but the analysis was adjusted (using STATA’s svy command) for the cluster sampling methodology. |
|  |  | (*e*) Describe any sensitivity analyses | NA | None conducted |
| Results | | | | |
| Participants | 13* | (a) Report numbers of individuals at each stage of study—eg numbers potentially eligible, examined for eligibility, confirmed eligible, included in the study, completing follow-up, and analysed | 10, 12 | Methods, statistical analysis sub-section, para 1: Only individuals with complete serological, infection and clinical data were included in the analysis.  Results, para 1: A total of 10,902 DBSs were analysed by MBA |
|  |  | (b) Give reasons for non-participation at each stage | NA |  |
|  |  | (c) Consider use of a flow diagram |  | Not required |
| Descriptive data | 14* | (a) Give characteristics of study participants (eg demographic, clinical, social) and information on exposures and potential confounders | 11 | Results, para 2 and table 2  Table of samples by age, median ages and gender proportions given |
|  |  | (b) Indicate number of participants with missing data for each variable of interest |  | Excluded if did not have all data available |
|  |  | (c) *Cohort study*—Summarise follow-up time (eg, average and total amount) | NA |  |
| Outcome data | 15* | *Cohort study*—Report numbers of outcome events or summary measures over time | *NA* |  |
|  |  | *Case-control study—*Report numbers in each exposure category, or summary measures of exposure | *NA* |  |
|  |  | *Cross-sectional study—*Report numbers of outcome events or summary measures | *NA* |  |
| Main results | 16 | (*a*) Give unadjusted estimates and, if applicable, confounder-adjusted estimates and their precision (eg, 95% confidence interval). Make clear which confounders were adjusted for and why they were included | 10-15 | Through-out the results section: Basic analysis is given as is required for this kind of study. |
|  |  | (*b*) Report category boundaries when continuous variables were categorized | 14-16 | Table 3 and 4 give details on EUs and TF at baseline. |
|  |  | (*c*) If relevant, consider translating estimates of relative risk into absolute risk for a meaningful time period | NA |  |

Continued on next page

| Other analyses | 17 | Report other analyses done—eg analyses of subgroups and interactions, and sensitivity analyses | 16 | Results, heterogeneity of data sub-section: DE and ICC |
| --- | --- | --- | --- | --- |
| Discussion | | | | |
| Key results | 18 | Summarise key results with reference to study objectives | 17  20 | Discussion, para 1: As evidenced in this study, elimination of trachoma as a public health problem does not equate to the absence of ocular Ct infection nor cessation in acquisition of anti-Ct antibodies.  Discussion, final para: Infection and in particular serological data provide useful insights into transmission dynamics. Even if an EU meets trachoma elimination targets, this may not reflect complete interruption of transmission of Ct infection. |
| Limitations | 19 | Discuss limitations of the study, taking into account sources of potential bias or imprecision. Discuss both direction and magnitude of any potential bias | 17  18 | Discussion, para 3: There are some concerns pooling can reduce the sensitivity of the test. Evidence suggests the impact would be minimal and likely to affect those individuals with lower ocular bacterial load, who are likely to be less important as drivers of transmission  Discussion, final para of pg 18: It is difficult to directly compare the seropositivity rates across studies because there is currently no agreed standard methodology for defining the threshold used to determine seropositivity. Another difficulty in interpreting the serological data is that antibodies to Pgp3 do not distinguish between urogenital and ocular infection, and Ct exposure could have occurred at birth through ocular or respiratory infection from a mother with genital Ct |
| Interpretation | 20 | Give a cautious overall interpretation of results considering objectives, limitations, multiplicity of analyses, results from similar studies, and other relevant evidence | 16-20 | As above |
| Generalisability | 21 | Discuss the generalisability (external validity) of the study results | 20 | Discussion, final para: Infection and in particular serological data provide useful insights into transmission dynamics. Even if an EU meets trachoma elimination targets, this may not reflect complete interruption of transmission of Ct infection. |
| Other information | |  | | |
| Funding | 22 | Give the source of funding and the role of the funders for the present study and, if applicable, for the original study on which the present article is based |  | Added to PLOS submission |

*Give information separately for cases and controls in case-control studies and, if applicable, for exposed and unexposed groups in cohort and cross-sectional studies.

**Note:** An Explanation and Elaboration article discusses each checklist item and gives methodological background and published examples of transparent reporting. The STROBE checklist is best used in conjunction with this article (freely available on the Web sites of PLoS Medicine at http://www.plosmedicine.org/, Annals of Internal Medicine at http://www.annals.org/, and Epidemiology at http://www.epidem.com/). Information on the STROBE Initiative is available at www.strobe-statement.org.
